# No Difference Among Inhaled Anesthetics on the Growth and Metastasis of Murine 4T1 Breast Cancers in a Mouse Model of Spontaneous Metastasis

**DOI:** 10.3389/fphar.2022.794109

**Published:** 2022-02-09

**Authors:** Qiuyue Liu, Ru Li, Jun Lin

**Affiliations:** ^1^ Department of Anesthesiology, Stony Brook University School of Medicine, Stony Brook, NY, United States; ^2^ Currently Department of Intensive Care Unit, Beijing Chest Hospital, Beijing Tuberculosis and Thoracic Tumor Research Institute, Capital Medical University, Beijing, China

**Keywords:** isoflurane, sevoflurane, desflurane, cell viability, migration, breast cancer, tumor growth, metastasis

## Abstract

**Objective:** This study evaluates the effect of the commonly used inhaled anesthetics isoflurane, sevoflurane, and desflurane on the viability and migration of murine 4T1 breast cancer cells, the growth, and lung metastasis in a syngenetic model of spontaneous metastasis.

**Methods:** The murine 4T1 breast cancer cells were exposed to isoflurane (2%), sevoflurane (3.6%), or desflurane (10.3%) for 3 h. Cell viability was measured using the MTT assay. The migratory capacity of 4T1 cells was assessed using a scratch assay after 24 h incubation. Female balb/c mice were subjected to orthotopic implantation of 4T1 cells under anesthesia with one of the inhaled anesthetics: 2% isoflurane, 3.6% sevoflurane, or 10.3% desflurane. Subsequently, resection of primary tumors was performed under the identical anesthetic used during implantation for 3 h. Three weeks later, the mice were euthanized to harvest lungs for *ex vivo* bioluminescent imaging and histological analysis. Blood was collected for serum cytokine assays by ELISA.

**Results:** There was no difference in cell viability among isoflurane, sevoflurane, desflurane, and control groups (*n* = 180 for each group, *P* = 0.648). Sevoflurane but not isoflurane or desflurane significantly increased the migration of 4T1 cells compared to the control group (*n* = 18, *P* = 0.024). There was no difference in the growth of the orthotopically implanted primary tumors (*n* = 12 for the isoflurane group, *n* = 11 for the sevoflurane group, and for the desflurane group, *P* = 0.879). Surgical dissection of primary tumors in mice under anesthesia with isoflurane, sevoflurane, or desflurane led to no difference in lung metastasis following surgery (*P* = 0.789). No significant difference was observed among isoflurane, sevoflurane, and desflurane groups in the serum levels of IL-6 (*P* = 0.284), CCL-1 (*P* = 0.591), MCP-1 (*P* = 0.135), and VEGF (*P* = 0.354).

**Conclusion:** Our study demonstrated that sevoflurane increased the migration of 4T1 breast cancer cells *in vitro*. Inhaled anesthetics isoflurane, sevoflurane, and desflurane had no difference on the growth of primary tumor and the lung metastasis of 4T1 cells in the mouse model of spontaneous metastasis with surgical removal of primary tumors.

## Introduction

Breast cancer is one of the most common malignancies in women and the second most frequently occurring newly diagnosed cancers worldwide (Wörmann, 2017). Surgical resection greatly improves the patient outcome (Ferlay et al., 2019), but tumor recurrence or metastasis after surgery is still the main cause of cancer patient death. The perioperative period carries many risks for cancer patients such that surgical procedures may disseminate cancer cells into the circulation and surrounding tissues (Camara et al., 2006). The number of circulating tumor cells has been shown correlating to the outcome of patients (Barbazan et al., 2014; Bortolini Silveira et al., 2021). The viability and motility of those cancer cells released from primary tumors may determine the spread and the development of clinical metastasis.

Inhaled anesthetics are routinely used for the maintenance of general anesthesia, and the choice of a particular anesthetic is at the discretion of the anesthesia provider. Isoflurane, sevoflurane, and desflurane are the most widely used inhaled anesthetics and have been suggested to influence the patient outcome following oncologic surgery (Buggy et al., 2015). Some retrospective studies have suggested that inhaled anesthetics may increase cancer recurrence, but not confirmed by other retrospective studies and a prospective clinical study (Enlund et al., 2014; Wigmore et al., 2016; Kim et al., 2017; Yoo et al., 2019; Sessler et al., 2019). Laboratory research has shown that inhaled anesthetics may change the microenvironment in healthy organs (Sakamoto et al., 2005) and alter mRNA expression in cancer cells (Jiao et al., 2018). It has also been shown that inhaled anesthetics promoted ovarian cancer cell migration and expression of metastasis-related genes and protein, which included VEGF-A, MMP-11, CXCR2, and TGF-β with a magnitude order of desflurane, sevoflurane, and isoflurane (Iwasaki et al., 2016). Our previous study found that sevoflurane was associated with more postoperative lung metastasis than intravenous anesthetic propofol in mouse models of spontaneous metastasis, of which the mechanism was linked to inflammatory cytokine IL-6 (Li et al., 2020). Thus, the difference of inhaled anesthetics on the cancer biology may lead to clinical significance.

No study has analyzed the difference among the commonly used inhaled anesthetics on the tumor growth and metastasis. The potential difference in inhaled anesthetics is important in evaluating the results of animal and human studies and selecting anesthetics in clinical studies or practice. Therefore, we hypothesized that inhaled anesthetics isoflurane, sevoflurane, and desflurane differentially affect the metastatic function of breast cancers at clinically relevant concentration. We tested our hypothesis in a preclinical mouse model of spontaneous metastasis using 4T1 cells as the primary endpoint and cellular functions of 4T-1 cells *in vitro* as secondary endpoints. Since IL-6 was associated with the promoting effect of sevoflurane on lung metastasis (Li et al., 2020), we measured the levels of IL-6 and other inflammatory cytokines including CCL-1, MCP-1, and VEGF as well.

## Methods

### Ethics Statement

All of the mice used in these experimental procedures were approved by the Institutional Animal Care and Use Committee (IACUC) at Stony Brook University (917821). Balb/c mice were purchased from the Jackson Laboratory (Bar Harbor, ME United States) and maintained in accordance with federal guidelines. Mice were housed in sterilized plastic cages under pathogen-free conditions (21–25°C, 12/12 light/dark cycle). Food and water were offered *ad libitum*. Mice were euthanized using CO_2_ overdose followed by cervical dislocation to ameliorate the suffering of mice.

### Test Gas Exposure

The treatment with different gases was conducted in a purpose-built 1.5 L airtight gas chamber equipped with inlet and outlet valves (Iwasaki et al., 2016). All gases were delivered to the gas chamber at a rate of 1 L/min and monitored using an anesthetic analyzer (POET IQ Anesthesia Gas Monitor, CRITICARE Systems ING) until the desired anesthetic concentrations were achieved. Then the chamber of gases was sealed and placed in an incubator at 37°C for the duration of 3 h. The experimental gases were air (medical grade) or one of the inhaled anesthetics in air: 2% isoflurane (Baxter, Deerfield, IL, United States), 3.6% sevoflurane (Baxter, Deerfield, United States), or 10.3% desflurane (Baxter, Deerfield, United States). The concentrations of the anesthetic gases are the equivalence of 1.7 minimum alveolar concentrations (MAC) in humans. After exposure, cells were returned to the normal cell culture incubator for further study.

### Cell Culture and Survival Assay

The murine breast cancer cell line 4T1-LUC was purchased from the American Type Culture Collection (Rockville, MD, United States) and cultured in RPMI 1640 supplemented with 10% FBS (Weene, l, United States), 100 U/ml penicillin, and 0.1 mg/ml streptomycin (Weene, l, United States) in 5% CO_2_ humidified atmosphere at 37°C. For the survival assay, cells were divided into a 96-well plate and incubated at 37°C for 24 h, and then treated with air (control) or one of the tested anesthetic gases in air for 3 h. Cell viability was measured using the MTT assay after 24 h incubation as previously described (Li et al., 2020). In brief, the culture medium was removed, and 100 µL MTT/medium solution(2.5 mg/ml) were added to each well and incubated for 3 h; then the medium was removed, and 100 µl aliquot of DMSO were added to each well to solubilize the formazan crystals. Absorbance was measured at 571 nm using a microplate reader (BioTek, Winooski, VT, United States). The percentage of cell viability was expressed relative to the control.

### Migration Assay

A wound healing assay was employed to evaluate the effects of isoflurane, sevoflurane, or desflurane on the cell migratory ability. The 4T1 cells were seeded at a density of 2 × 10^6^ cells/well in 6-well plates and incubated for 12 h at 37°C to allow adherence to take place. The scratches were then made using a 100-µl yellow tip (time 0), transferred to the low-serum culture medium, and treated with 2% isoflurane, 3.6% sevoflurane, or 10.3% desflurane for 3 h. The distances of migrating cells were measured from pictures (five fields) taken at 24 h after the initial wound, and the distance of each measurement was calculated by using ImageJ (NIH, Bethesda, MD, United States). Each experiment was independently repeated at least three times.

### Animal Models and Surgery

Female balb/c mice in each group were subjected to orthotopic implantation of 4T1 cells (2 × 10^5^ cells per mice) in the mammary fat pad. Implantations were conducted with one of the inhaled anesthetics (2% isoflurane, 3.6% sevoflurane, or 10.3% desflurane) within 10 min. The growth of 4T1 tumors was monitored by non-invasive bioluminescent imaging (IVIS Lumina III, PerkinElmer, Waltham, MA). The volume of tumors was measured using a caliper every week and calculated using formula V = (Width^2^ × Length) × 2^–1^. When the volume of the primary tumor reached around 500 mm^3^, the primary tumors were dissected under the identical anesthetic used for cancer cell implantation, and the anesthesia was maintained for 3 h. During surgery, the delivery of inhaled anesthetics was maintained using a SomnoSuite Rodent Anesthesia System (Kent Scientific Corporation, Torrington, CT, United States), and the oxygen saturation and heart rate were monitored by using the PhysioSuite (Kent Scientific Corporation, Torrington, CT, United States) with a pulse oximeter. The mice were placed on the warming pad for temperature control with the SomnoSuite. After surgery, lung metastasis was monitored by using non-invasive bioluminescent imaging after 3 weeks. Three weeks later, the mice were euthanized to harvest lungs for *ex vivo* bioluminescent imaging and histological analysis. Blood was collected for the serum cytokine assay.

### Hematoxylin and Eosin Staining and Nodule Counting

Harvested mouse lungs were rinsed in PBS buffer to remove the blood and then fixed in 4% paraformaldehyde overnight at 4°C. Tissues were embedded in paraffin, and a sampling of sections was taken across the lung as follows: two consecutive 5 μm sections were taken, and then a number of consecutive 5 μm sections were discarded (typically 20–40 depending on the size of the tumor nodules) before collecting another two consecutive 5 μm sections. This process was repeated along with the entire lung. The consecutive sections were then stained using H&E, and metastatic nodules were counted on each H&E paraffin section using a phase contrast microscope. The sum of microscopic counting was taken as the final number of lung metastatic nodules.

### ELISA Assay

Mouse serum was subjected to IL-6, CCL-1, MCP-1, and VEGF ELISA assays according to the manufacturer instructions (R&D Systems, Minneapolis, MN, United States). The concentrations of IL-6, CCL-1, MCP-1, and VEGF in serum were calculated according to the volume of serum.

### Statistical Analysis

For the animal experiment, 11 mice per group would provide 80% power to detect 30% difference in the total burden of metastasis among three groups treated with inhaled anesthetics at the α level of 0.05, based on a sample size calculation using JMP by SAS (version 10). Statistical analysis was performed using GraphPad Prism 7.0. All the values were expressed as means ± SD. The data were analyzed using ANOVA. Differences were considered significant at *p* < 0.05.

## Results

### Inhaled Anesthetics Have no Significant Effect on the Viability of 4T1-Luc Cells

**FIGURE 1:**
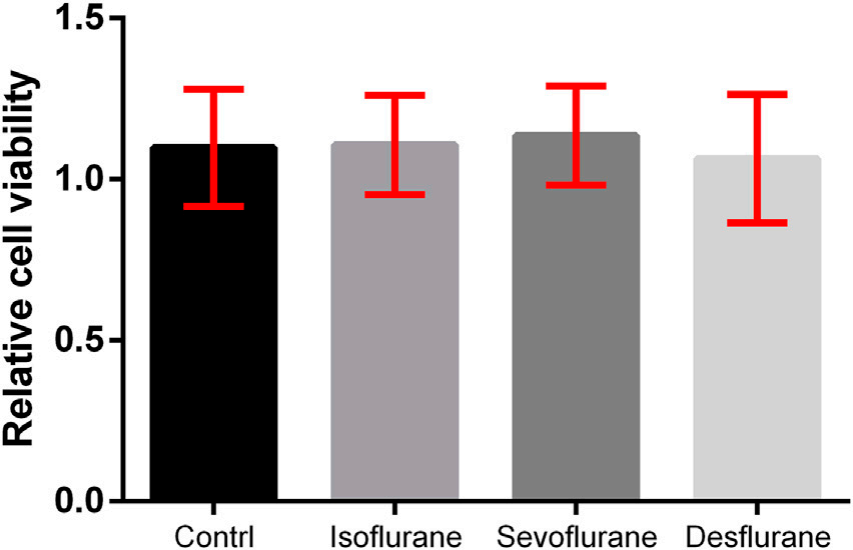
Effect of inhaled anesthetics on the viability of 4T1 cells *in vitro*. The 4T1 cells were treated with air (control), 2% isoflurane, 3.6% sevoflurane, or 10.3% desflurane for 3 h. Cell viability was determined using the MTT assay. There was no significant difference between the four groups (*n* = 180, *P* = 0.648). Isoflurane vs. control, *P* = 0.684; sevoflurane vs. control, *P* = 0.541; desflurane vs. control, *P* = 0.363; one-way ANOVA + Tukey’s multiple comparisons test.

The 4T1 LUC cells were treated with air, 2% isoflurane, 3.6% sevoflurane, or 10.3% desflurane (*n* = 180 for each group). The viability (%) of 4T1 cells treated with inhaled anesthetics for 3 h and the statistical differences between groups are illustrated in Figure 1. There is no significant difference in the viability of the 4T1 cell among the control, isoflurane, sevoflurane, and desflurane groups (*n* = 180 for each group, *P* = 0.648).

### Sevoflurane Promotes the Migration of 4T1-Luc Cells

**FIGURE 2:**
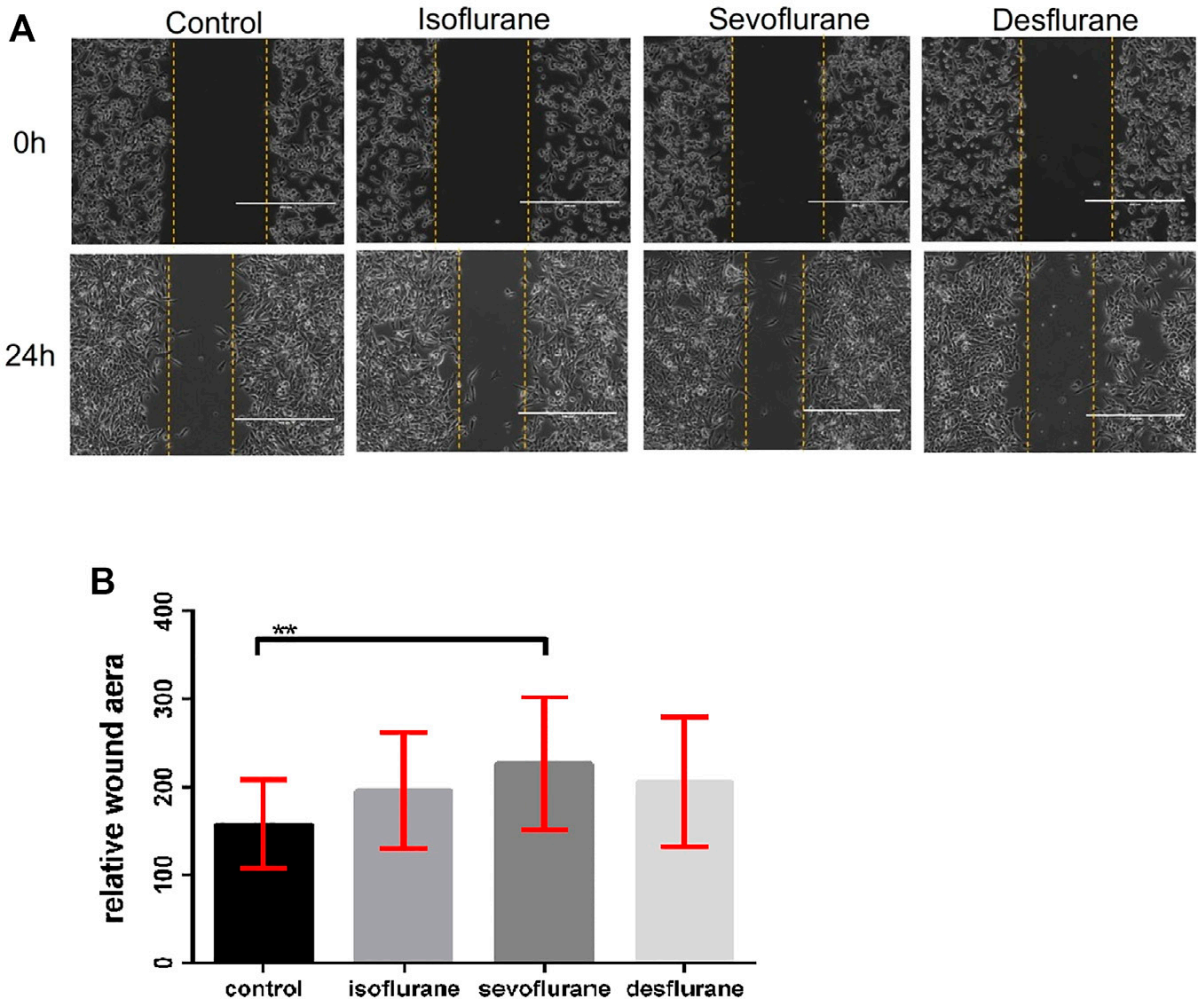
Effect of inhaled anesthetics on the migratory capacity of 4T1 cells *in vitro*. **(A)** The 4T1 cells were wounded by a sterile pipette tip to create a cell-free path, and then they were treated with air (control), 2% isoflflurane, 3.6% sevoflflurane, or 10.3% desflflurane for 3 h (*n* = 18 for each group). **(B)** Relative wound distance was measured for statistical analysis. The differences between the control group and the sevoflflurane group was signifificant (*P* = 0.024). There was no signifificant difference between isoflflurane or desflflurane and control group. Isoflflurane vs. control, *P* = 0.153; desflflurane vs. control, *P* = 0.465; one-way ANOVA + Tukey’s multiple comparisons test.

Wound healing assays were used to evaluate the effects of inhaled anesthetics on cell migration. There is a tendency that the gap closures were accelerated by treatment with 2% isoflurane, 3.6% sevoflurane, or 10.3% desflurane compared to the control at 24 h post-exposure (*n* = 18 for each group, Figure 2). Only sevoflurane significantly affected the migration of 4T1 cells in comparison with the control group (*P* = 0.024).

### Effect of Inhaled Anesthetics on Lung Metastases in 4T1 Murine Cancer Mouse Model

**FIGURE 3:**
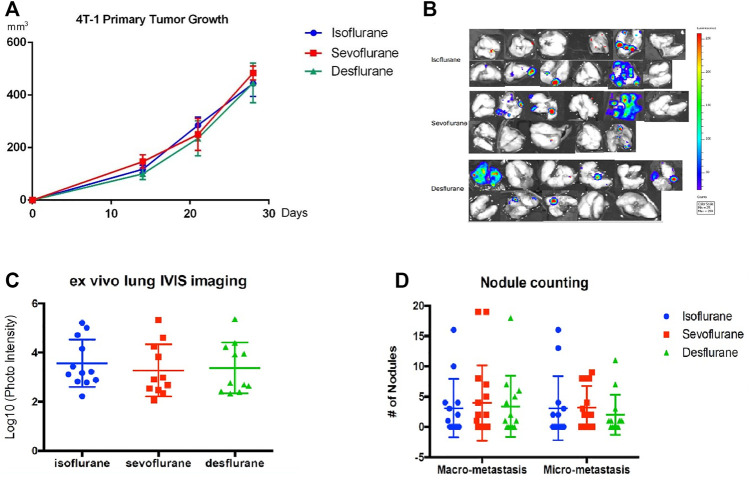
No significant difference in the lung metastasis following mastectomy with different inhaled anesthetics. Mice bearing primary tumors were generated by orthotopical implantation with the luciferase-tagged murine 4T1 breast cancer cells in the mammary fat pads of balb/c mice (*n* = 12 for isoflurane group, *n* = 11 for sevoflurane group, and *n* = 11 for desflurane group). Surgical dissection of primary tumor with 2% isoflurane, 3.6% sevoflurane, or 10.3% desflurane. Mastectomy was performed in mice models, and lung metastases were evaluated 3 weeks after surgery. **(A)** There was no difference in the primary tumor volumes among isoflurane, sevoflurane, or desflurane groups (isoflurane vs. sevoflurane, *P* = 0.901; isoflurane vs. desflurane, *P* = 0.847; sevoflurane vs. desflurane *P* = 0.645; one-way ANOVA + Tukey’s multiple comparisons test), **(B)** *ex vivo* lung bioluminescent imaging, and **(C)** photon intensity of them showed no significant difference in lung metastasis among isoflurane, sevoflurane, or desflurane groups (isoflurane vs. sevoflurane, *P* = 0.778; isoflurane vs. desflurane, *P* = 0.899; sevoflurane vs. desflurane, *P* = 0.971). **(D)** The examination of number and size of metastatic nodules showed no significant difference among isoflurane, sevoflurane, or desflurane groups (isoflurane vs. sevoflurane, *P* = 0.996; isoflurane vs. desflurane *P* = 0.993; sevoflurane vs. desflurane, *P* = 0.986).

The implantation of murine 4T1-Luc cells stably expressing luciferase in the unilateral mammary fat pad of balb/c mice was carried out under one of the inhaled anesthetics: 2% isoflurane, 3.6% sevoflurane, or 10.3% desflurane (*n* = 12 for isoflurane group, *n* = 11 for sevoflurane group, and *n* = 11 for desflurane group). Surgical dissection was conducted under the same anesthetic for 3 h when the volume of the primary tumor reached around 500 mm^3^. There is no significant difference in primary tumor volumes in 3 groups (*P* = 0.789, Figure 3A). Three weeks after surgical removal of the primary tumor, no significant difference in the burden of lung metastasis was observed in the mice receiving different anesthetics (Figure 3B), which was confirmed by histology analysis of nodule counts (Figures 3C,D).

**FIGURE 4:**
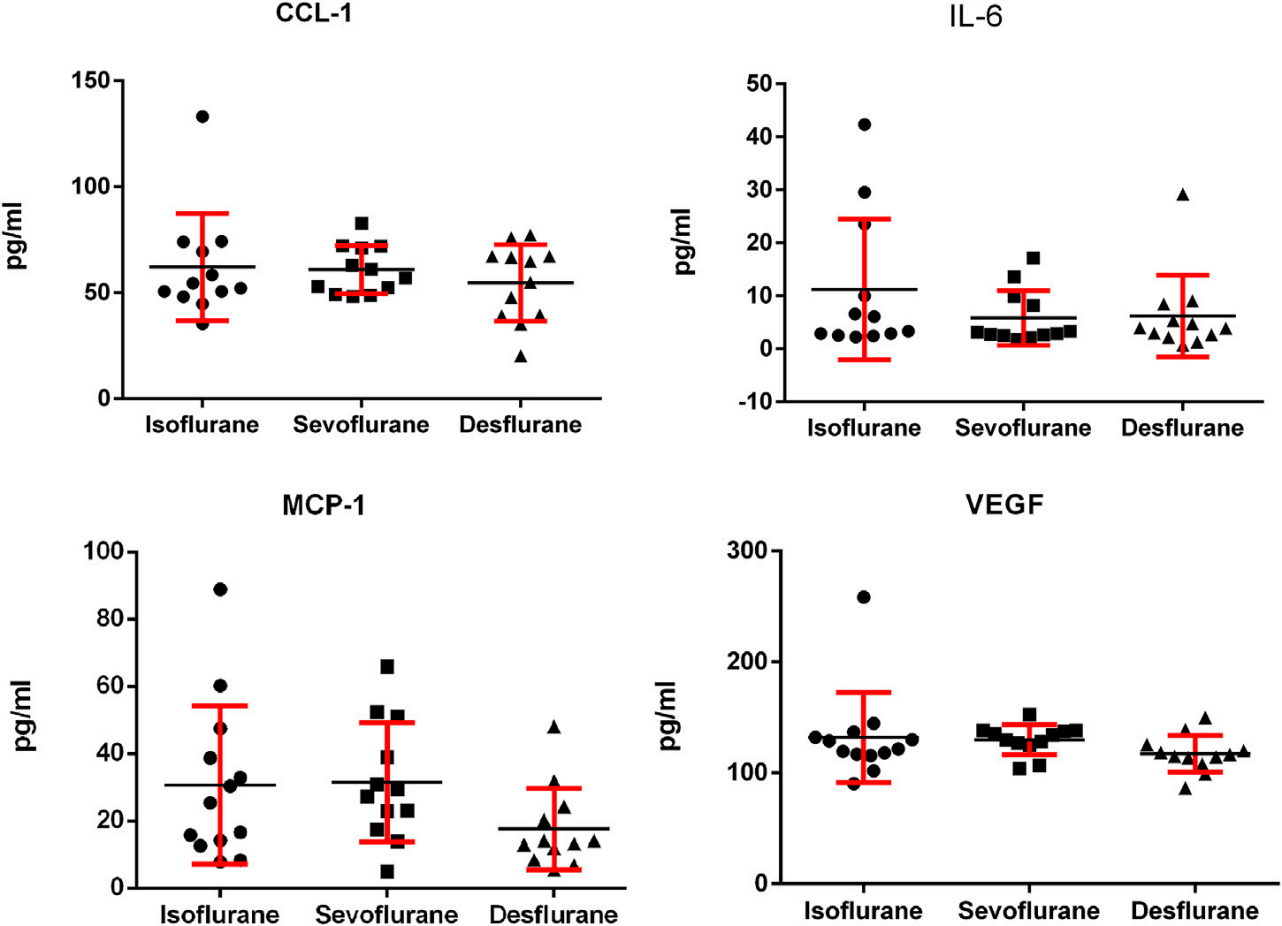
Serum levels of IL-6, MCP-1, CCL-1, and VEGF in the murine 4T1 breast tumor bearing mice 3 weeks following mastectomy with isoflurane, sevoflurane, or desflurane. There was no significant difference in serum levels of IL-6, MCP-1, CCL-1, and VEGF among 3 groups (*n* = 12 for isoflurane group, *n* = 11 for sevoflurane group, and *n* = 11 for desflurane group; one-way ANOVA + Tukey’s multiple comparisons test). IL-6: isoflurane vs. sevoflurane, *P* = 0.508; isoflurane vs. desflurane, *P* = 0.317; sevoflurane vs. desflurane, *P* = 0.445. MCP-1: isoflurane vs. sevoflurane *P* = 0.212; isoflurane vs. desflurane, *P* = 0.134; sevoflurane vs. desflurane, *P* = 0.106. CCL-1: isoflurane vs. sevoflurane *P* = 0.504; isoflurane vs. desflurane, *P* = 0.334; sevoflurane vs. desflurane, *P* = 0.590. VEGF: isoflurane vs. sevoflurane, *P* = 0.354; isoflurane vs. desflurane, *P* = 0.457; sevoflurane vs. desflurane, *P* = 0.389.

We analyzed the effect of inhaled anesthetics on the serum levels of inflammatory cytokines. No significant difference was observed among isoflurane, sevoflurane, and desflurane groups in serum levels of IL-6, CCL-1, MCP-1, and VEGF (Figure 4). The desflurane group has a trend of lower MCP-1 than the other two anesthetics, but it was not statistically significant.

## Discussion

The role of anesthesia in patient outcome remains to be defined. One question is whether there is a difference among inhaled anesthetics on cancer cell biology that may affect the patient outcome. Our results suggest there is no significant difference in metastatic functions of murine breast cancers and support the practice that groups all inhaled anesthetics together in retrospective clinical studies. Our data are also informative to the animal studies involving the use of general anesthetics. The limitation of our study should be noted; however, as our results were obtained from murine breast cancer, it may not be applicable to all other cancer types or human cancers.

A significant finding of this study is that sevoflurane at the clinically relevant concentration increased migration of 4T1-luc breast cancer cells *in vitro*. Migration is the basic biological process that is essential for tumor cells to metastasize. There are reports showing inhaled anesthetics enhance the malignancy of cancer cells by different mechanisms. Sevoflurane increased cell viability, migration, and chemoresistance of renal carcinoma by upregulating TGF-βRII and OPN (Ciechanowicz et al., 2018). Sevoflurane increased the migration and colony formation of human glioblastoma cells *via* the expression of CD44 (Lai et al., 2019). Sevoflurane promoted the proliferation and migration of immortalized cervical cancer cells through the activation of phosphatidylinositide 3-kinase/AKT- and ERK1/2-signaling pathway activation (Zhang et al., 2020). Sevoflurane increased migrations of breast cancer estrogen receptor (ER)-positive MCF7 cells and ER-negative MDA-MB- 231 cells (Ecimovic et al., 2013). Isoflurane activated the expression of HIF-1α and its downstream effectors in prostate PC3 cancer cells, leading to increased migration (Huang et al., 2014). In addition, isoflurane increased the levels of HIF-1α, HIF-2α, and VEGF in primary renal cell carcinoma (Benzonana et al., 2013). Indeed, we found that sevoflurane enhanced 4T1 cell migration significantly, but we did not observe any significant effect of sevoflurane on the viability of 4T1 cells. We found a tendency of increase in migration with isoflurane and desflurane. All three inhaled anesthetics did not have a significant effect on viability. Thus, the effects of inhaled anesthetics on the biology of cancer cells appear to vary among types of cancers.

A variety of factors regulate cancer cell migration including matrix-degrading enzymes and cell–cell adhesion molecules. As the change of cell viability and migration *in vitro* do not always translate to the effect of tumor growth and metastasis *in vivo* (Li et al., 2020), which is more clinically relevant to our hypothesis, we elected to analyze the effect of the inhaled anesthetics in a mouse model of spontaneous metastasis. This orthotopically implanted model is a preclinical model with a high clinical predictive value (Shan et al., 2005; Bailey-Downs et al., 2014). Surgery to remove primary tumor was incorporated to closely mimic the clinical scenario. Inflammatory cytokines such as IL-6, CCL-1, MCP-1, and VEGF play a vital role in cancer progression and metastasis (Kaplan et al., 2005; Gril et al., 2018; Li et al., 2020). We have shown, in our previous report, that sevoflurane increased the activity of the IL-6 pathway, leading to more lung metastatic burden than propofol (Li et al., 2020). In this study, we did not observe any significant difference of primary tumor growth and the lung metastasis in the mice receiving different inhaled anesthetics after surgery, nor in the levels of pro-inflammatory cytokines (IL-6, MCP-1, CCL-1, or VEGF). The desflurane group had a trend of lower MCP-1 than the other two anesthetics, and it was however not statistically significant. Therefore, we conclude that there is no significant difference among inhaled anesthetics on the primary tumor growth and postoperative metastasis in our models. Taken together, our data and literature show that inhaled anesthetics affect cancer cells *in vitro* differently but suggest no significant difference in the primary tumor growth and the metastasis *in vivo*.

## Limitation

One limitation of our study is to evaluate the possibility of difference in inhaled anesthetics on tumor metastasis in one cell line and one animal model. Second, different doses or time courses may produce more anesthetic effects on cancer cells, even the non-specific effects of volatile drugs. Another limitation of this study is the relatively small sample size to detect a small change, and that populations are more at risk of such obese mice have not been studied.

## Data Availability

The original contributions presented in the study are included in the article/Supplementary Material; further inquiries can be directed to the corresponding author.

## Abbreviations

CCL-1, chemokine ligand-1; CXCR-2, CXC chemokine receptor type-2; ELISA, enzyme-linked immunosorbent assay; HIF, hypoxia-inducible factor; IL-6, interleukin-6; OPN, osteopontin; MCP-1, mono-cyte chemotactic protein-1; MMP-11, matrix metallopeptidase-11; TGF-β, transforming growth factor-β; TGF-βRII, transforming growth factor-β receptor type II; VEGF-A, vascular endothelial growth factor-A.
